# Technological Solutions for Cardiac Surgery in the Elderly

**DOI:** 10.5041/RMMJ.10120

**Published:** 2013-07-25

**Authors:** Rony-Reuven Nir, Gil Bolotin

**Affiliations:** Cardiovascular Surgery, Rambam Health Care Campus and the Faculty of Medicine, Technion – Israel Institute of Technology, Haifa, Israel

**Keywords:** Cardiac surgery, intraoperative epiaortic ultrasound, octogenarians, off-pump coronary artery bypass grafting (CABG), proximal anastomosis device, transcatheter aortic valve implantation (TAVI)

## Abstract

The current review addresses contemporary technological advances in cardiac surgery performed on octogenarian patients, namely off-pump coronary artery bypass grafting (CABG), proximal anastomosis device, routine use of intraoperative epiaortic ultrasound, proximal anastomosis without clamping, transcatheter aortic valve implantation (TAVI), and brain protection during cardiac surgery.

## GENERAL CHARACTERISTICS

In a thorough investigation encompassing 22 centers, Alexander et al. evaluated the characteristics and outcomes of 4,743 octogenarian patients undergoing cardiac surgery, as compared to 63,021 younger patients.^1^ Main findings suggested that: 1) Octogenarians undergoing cardiac surgery had fewer co-morbid illnesses but higher disease severity and surgical urgency than younger patients; 2) Octogenarians had significantly higher in-hospital mortality after cardiac surgery than younger patients: coronary artery bypass grafting (CABG) only: 8.1% versus 3.0%, CABG/aortic valve replacement (AVR): 10.1% versus 7.9%, CABG/mitral valve replacement (MVR): 19.6% versus 12.2%; 3) Octogenarians had twice the incidence of postoperative stroke and renal failure; 4) The preoperative clinical factors predicting CABG mortality in the very elderly were quite similar to those for younger patients, with age, emergency surgery, and prior CABG being the powerful predictors of outcome in both age categories; 5) Importantly, elderly patients without significant co-morbidities had in-hospital mortality rates of 4.2% after CABG, 7% after CABG with aortic valve replacement (CABG/AVR), and 18.2% after CABG with mitral valve replacement (CABG/MVR).

## OFF-PUMP CABG IN OCTOGENARIANS

Specific patients seem to benefit from off-pump CABG compared with conventional CABG with cardiopulmonary bypass. The effectiveness of off-pump procedures is still debated in elderly patients undergoing isolated CABG operations.

Ricci et al. investigated the potential benefits of coronary artery bypass grafting without cardiopulmonary bypass (CPB) for octogenarians.^2^ They studied 269 octogenarians who underwent coronary artery bypass grafting, of whom 172 had the operation with CPB and 97 without CPB (off-pump group). Findings suggested that: 1) A greater proportion of reoperations was observed in the off-pump cohort (16 of 97 (16.5%)) compared with the CPB cohort (8 of 172 (4.7%)) (*P* = 0.002); 2) Freedom from postoperative complications was higher in the off-pump group than in the CPB group (83 of 97 (85.6%) versus 129 of 172 (75%), *P* = 0.04); 3) The incidence of stroke was 0% in the off-pump cohort compared with 9.3% (16 of 172) in the CPB cohort (*P* < 0.0005). These findings suggest that patients 80 years of age and older undergoing off-pump CABG can experience significantly lower rates of perioperative stroke and overall complications compared with those undergoing the same procedure with CPB.

In the same vein, Demaria et al. studied 125 patients older than 80 years of age who were operated for isolated myocardial revascularization (63 using CPB and 62 with off-pump coronary artery bypass (OPCAB)) over a 5-year period (1995–1999).^3^ These groups were comparable in terms of: 1) Preoperative co-morbidities; 2) Mean left ventricular ejection fraction (54.5% ± 15.3% in the CPB group and 50.9% ± 13.5% in the OPCAB group); and 3) The mean number of distal anastomoses per patient (2.9 in the CPB group and 2.6 in the OPCAB group). The majority of patients in both groups had unstable angina and were operated on an urgent basis. The main results were that: 1) The operative mortality was 15.9% in the CPB group and 4.8% in the OPCAB group (*P* = 0.04); 2) There were 4 postoperative strokes (6.3%) in the CPB group and none (0%) in the OPCAB group (*P* = 0.04); 3) The percentage of patients transfused was 92.1% in the CPB group and 72.6% in the OPCAB group (*P* < 0.01); 4) Postoperative myocardial infarction occurred in 11.3% in the CPB group and 14.5% in the OPCAB group (*P* = NS); 5) The type of surgery (CPB or OPCAB) was an independent predictor of operative mortality and stroke (*P* = 0.0375); 6) The odds ratio (OR) indicated that operative mortality and stroke occur 4 times (OR = 4.171) more often in CPB patients than in OPCAB patients; and 7) Follow-up showed no significant difference between the two groups in terms of cardiac events and mortality. These findings may indicate that a benefit of OPCAB in terms of operative mortality and stroke exists for octogenarian patients when compared with CPB.

LaPar et al. examined 1,993 elderly patients (age ≥ 80 years) who underwent isolated, primary CABG operations at 16 centers from 2003 to 2008.^4^ Patients were stratified into two groups: Conventional coronary artery bypass (*n* = 1,589, age = 82.5 ± 2.4 years) and off-pump bypass (*n* = 404, age = 83.0 ± 2.4 years). The main findings were that patients undergoing off-pump bypass grafting: 1) Were marginally older (*P* = 0.001); 2) Had higher rates of preoperative atrial fibrillation (14.6% versus 10.0%, *P* = 0.01) and New York Heart Association (NYHA) class IV heart failure (29.7% versus 21.1%, *P* < 0.001) than did those having conventional CABG; 3) Other patient risk factors and operative variables, including Society of Thoracic Surgeons predicted risk of mortality, were similar in both groups. Compared with off-pump bypass, conventional coronary bypass incurred 1) Higher blood transfusion rates (2.0 ± 1.7 units versus 1.6 ± 1.9 units, *P* = 0.05); 2) More postoperative atrial fibrillation (28.4% versus 21.5%, *P* = 0.003); 3) Prolonged ventilation (14.7% versus 11.4%, *P* = 0.05); and 4) Major complications (20.1% versus 15.6%, *P* = 0.04). Notably, postoperative stroke (2.6% versus 1.7%), renal failure (8.1% versus 6.2%), and postoperative length of stay were comparable. In spite of more complications in patients having conventional bypass, operative mortality and hospital costs were similar to those of patients having off-pump procedures. These observations may indicate that CABG procedures among octogenarians are safe and effective; off-pump CABG yields shorter postoperative ventilation but equivalent mortality to conventional coronary artery bypass. Finally, as off-pump CABG was associated with a reduction in the composite incidence of major complications, it should be considered an adequate alternative to conventional CABG for myocardial revascularization in elderly patients.

## PROXIMAL ANASTOMOSIS DEVICE

Manipulation of the ascending aorta during cardiac surgery is one of the leading causes of embolic events and postoperative stroke; atheromatous disease of the ascending aorta is more prevalent in the elderly population.^5^,^6^ Evolving technologies of proximal anastomosis devices tested the ability to serve the goal of surgeons to minimize manipulation of the ascending aorta during surgical myocardial revascularization, as emboli generated during cardiac surgery have been associated with aortic clamping and manipulation. At first, proximal anastomotic devices were thought to be less traumatic by eliminating partial clamping, potentially resulting in fewer adverse outcomes; however, their use has ceased due to the accumulation of substantial discouraging evidence.

In 2004, Martens et al. compared the amount of debris released using intra-aortic filtration and the clinical outcomes between conventionally hand-sewn and automated proximal anastomoses.^5^ This comparison was carried out through the investigation of 77 patients undergoing primary CABG with cardiopulmonary bypass in a prospective randomized study. Patients were assigned to the anastomotic device Group I (Symmetry Aortic Connector, St. Jude Medical, Inc., St. Paul, MN, USA; *n* = 39) or the conventional hand-sewn anastomosis control Group II (*n* = 38). Proximal anastomoses were performed before cardiopulmonary bypass in both groups. Intra-aortic Filter 1 (EMBOL-X, Edwards Lifesciences LLC, Irvine, CA, USA) was deployed prior to partial clamping or puncturing the aorta for device application and removed after the proximal anastomosis was completed. Prior to cross-clamp removal, a second filter was inserted (Filter 2). A core laboratory performed quantitative and histologic analyses of the debris captured. Martens et al. demonstrated that preoperative variables and risk factors were not significantly different between Groups I and II (European System for Cardiac Operative Risk Evaluation (EuroSCORE) 3.9 ± 2.6 versus 4.2 ± 2.5, respectively). Filter analyses showed no significant difference between Groups I and II in Filter 1 or 2 for either surface area of particles or total number of particles. A decrease was observed between Filters 1 and 2 in both groups for surface area of particles (Group I: 18.5 ± 23.8 mm^2^ versus 10.7 ± 16.3 mm^2^, *P* = 0.017; Group II: 15.0 ± 15.4 mm^2^ versus 6.9 ± 6.5 mm^2^, *P* = 0.004), and for total number of particles in Group II (8.6 ± 3.7 versus 7.1 ± 2.4, *P* = 0.023). No significant differences were observed between Group I (device) and Group II (control) outcomes for myocardial infarction, neurocognitive deficit, stroke, length of stay, graft occlusion, or mortality. These findings pointed to the facts that 1) The application of proximal aortic connectors without partial clamping does not reduce particulate emboli or affect clinical outcomes compared with conventional anastomoses; and 2) Cross-clamping during cardiopulmonary bypass produces less particulate debris than conventional or automated proximal anastomoses performed off-pump—suggesting the anastomotic process was a major source of emboli.

In keeping with this, Bergsland et al. explored the patency in saphenous vein CABG in which the proximal anastomoses were performed with automatic connector devices or with a traditional suture technique on 46 patients who underwent OPCAB, using one thoracic graft and one or more saphenous vein grafts.^6^ Grafts were attached to the aorta with a Symmetry connector in 23 patients, and partial occlusion of the aorta and sutured anastomoses were used in 23 other patients. Angiography was repeated after 3–5 months. Bergsland et al. showed that 1) Intraoperative graft patency did not differ between the two groups; 2) Vein graft patency decreased to 50% in the Symmetry group, whereas it was 90% in the suture group (*P* = 0.01); and 3) Twenty-five percent of the Symmetry grafts had significant stenosis in the connector. These observations stressed the fact that saphenous vein grafts anastomosed to the aorta with the Symmetry proximal connector had low intermediate patency compared with those with traditionally sutured anastomoses. Importantly, the authors discouraged the routine use of this device in coronary artery bypass operations.

Although clinical practice inclined to reject the use of these devices, other studies reported a positive experience with advanced devices; Diegeler et al. compared the patency rate of the saphenous vein coronary bypass grafts in which the proximal anastomoses were performed with second-generation automatic connector devices to the suture technique.^7^ This was examined in 86 patients who underwent CABG with at least one vein graft anastomosed to the ascending aorta with the Symmetry G2 connector. Diegeler et al. reported that 1) Eighty patients had at least one connector successfully implanted; 2) Freedom from cardiac mortality, myocardial infarction, and target vessel reintervention was 72/80 (90%); 3) Six patients underwent a target vessel reintervention on the connector grafts; 4) Six-month (mean 193 ± 36 days) angiography patency rates for the connector grafts were 72/81 (88.89%), 37/40 (92.5%) in sutured grafts, and 60/62 (96.8%) in arterial grafts. The authors concluded that saphenous vein grafts anastomosed to the aorta with the Symmetry G2 connector had early and mid-term patency rates comparable to the conventional sutured anastomoses and that these results supported the efficiency of the second generation of symmetry aortic connectors.

## ROUTINE USE OF INTRAOPERATIVE EPIAORTIC ULTRASOUND

Atheroma release from the ascending aorta and proximal arch is a key reason for stroke and neurological injury following cardiac surgery.^8^ The precise discovery of atheroma before aortic manipulation is required to allow surgical strategies to decrease the risk of embolization. The conventional technique for atheroma discovery is manual palpation by the surgeon—a technique that misses ∼50% of the atheroma lesions, as the soft, non-calcified lesions offer little resistance to the surgeon’s fingers. Transesophageal echocardiography is commonly used in cardiac surgery, but the interposition of the bronchus between the aorta and the esophagus causes an ultrasound “blind spot” in the ascending aorta and proximal arch, such that it does not offer improved detection compared to manual palpation. Accurate detection of atheroma requires direct ultrasound assessment using epiaortic scanning, with a high-frequency, linear-array probe. This allows the surgeon to assess and localize any atheroma correctly.

Yamaguchi et al. explored the efficacy of intraoperative epiaortic ultrasound scanning (EAS) for preventing cerebral emboli following CABG by evaluating the ascending aorta in 909 consecutive CABG patients.^9^ The ascending aorta was manipulated only if the scanning documented more than 3 mm of atheromatous thickness or plaque; 196 patients (21.6%) underwent off-pump CABG using composite grafts (85 cases, 9.4%) or *in situ* grafts (111 cases, 12.2%) with no aortic manipulation. The ascending aorta was confirmed to be free from significant atheromatous plaque by the EAS in 713 patients (78.4%). On-pump CABG was performed using aortic cannulation and total aortic clamping in 429 patients (47.2%). Off-pump CABG with aortocoronary bypass grafts was performed using side-bite aortic clamping in 165 cases (18.2%) or the other anastomotic devices in 63 cases (6.9%). Results demonstrated that five hospital deaths occurred (0.6%), but no postoperative strokes. Postoperative coronary angiography revealed 98.8% (1,659/1,680) patency of the bypassed grafts. These findings suggest that the application of aortic clamping or cardiopulmonary bypass was not a risk factor of cerebral emboli when the ascending aorta was evaluated using the EAS. Furthermore, the application of aortic clamping with free grafts may provide eligible bypass graft patterns, leading to sufficient graft patency.

## PROXIMAL ANASTOMOSIS WITHOUT CLAMPING

Avoidance of manipulation of diseased ascending aorta has been shown to be associated with a reduced risk of postoperative stroke after OPCAB—an extremely desired outcome in the cardiovascular surgery setting where postoperative stroke is the Achilles’ heel of CABG compared with percutaneous coronary intervention. The use of the Heartstring device (Guidant, Indianapolis, IN, USA) to accomplish a proximal aortic anastomosis without aortic clamping has been suggested in such patients.

Biancari et al. addressed the use of the Heartstring anastomotic device in 19 patients with diseased, calcified ascending aorta who underwent OPCAB.^10^ The diagnosis of diseased ascending aorta was made intraoperatively by epiaortic ultrasound scanning. Biancari et al. demonstrated that 18 vein grafts and three radial artery grafts had been successfully anastomosed to the ascending aorta by employing the Heartstring device. The breaking of eight seals occurred during insertion. One patient (5.2%) had a stroke 2 days after urgent OPCAB. These observations suggest that the use of the Heartstring anastomotic device may be advantageous in high-risk patients with diseased ascending aorta requiring a prompt myocardial revascularization, whenever there is a place to insert this device safely into the ascending aorta.

A recent meta-analysis addressed the efficacy of the Heartstring proximal anastomotic device to reduce the risk of postoperative stroke after OPCAB.^11^ A total of 819 patients were enrolled from eight studies; six of them suffered postoperative stroke. Cumulative analysis showed a pooled rate of immediate postoperative stroke after OPCAB with the use of Heartstring of 1.9% (95% confidence interval (CI) 0.8–4.5). Sensitivity analysis including the only three studies evaluating patients with diseased ascending aorta as detected at epiaortic ultrasound showed that a pooled rate of stroke was 3.2% (95% CI 0.8–11.9). Six studies reported on immediate postoperative mortality, and the pooled mortality rate was 1.9% (95% CI 0.1–3.4). The results of this meta-analysis suggest that, on the one hand, the risk of stroke after OPCAB may not be markedly reduced by the use of Heartstring device; on the other hand, a rather low rate of stroke was observed among patients with diseased ascending aorta, indicating its potential value in these patients. Since the majority of the analyzed studies included in this meta-analysis were of poor methodological quality, properly conducted prospective studies are needed to get more conclusive results on the safety and efficacy of the Heartstring anastomosis device.

## TRANSCATHETER AORTIC VALVE IMPLANTATION (TAVI)

### Randomized studies (PARTNER I, II)

Transcatheter aortic valve implantation (TAVI) is an alternative option for patients with severe aortic stenosis (AS) who are classified as high-risk patients or patients not eligible for conventional aortic valve surgery. Quality-of-life (QOL) is a critical measure of effectiveness of TAVI in this patient population. Two major studies paved the way to the increasing clinical use of TAVI.^12^,^13^

Many patients with severe aortic stenosis and coexisting conditions are not candidates for surgical replacement of the aortic valve; this motivated Leon et al. to randomly assign 358 patients with severe aortic stenosis, whom surgeons considered not to be suitable candidates for surgery, to standard therapy or transfemoral transcatheter implantation of a balloon-expandable bovine pericardial valve.^12^ The primary end-point was the rate of death from any cause. Leon et al. found that 1) at 1 year, the rate of death from any cause was 30.7% with TAVI, as compared with 50.7% with standard therapy (hazard ratio with TAVI, 0.55, 95% CI 0.40–0.74, *P* < 0.001); 2) the rate of the composite end-point of death from any cause or repeat hospitalization was 42.5% with TAVI as compared with 71.6% with standard therapy (hazard ratio 0.46, 95% CI 0.35–0.59, *P* < 0.001); 3) among survivors at 1 year, the rate of cardiac symptoms (New York Heart Association class III or IV) was lower among patients who had undergone TAVI than among those who had received standard therapy (25.2% versus 58.0%, *P* < 0.001); 4) at 30 days, TAVI, as compared with standard therapy, was associated with a higher incidence of major strokes (5.0% versus 1.1%, *P* = 0.06) and major vascular complications (16.2% versus 1.1%, *P* < 0.001); and 5) in the year after TAVI, there was no deterioration in the functioning of the bioprosthetic valve, as assessed by evidence of stenosis or regurgitation on an echocardiogram. These pivotal findings indicated that in patients with severe aortic stenosis who were not suitable candidates for surgery, TAVI, as compared with standard therapy, significantly reduced the rates of death from any cause, the composite end-point of death from any cause or repeat hospitalization, and cardiac symptoms, despite the higher incidence of major strokes and major vascular events.

Smith et al. addressed these procedures in 699 randomly assigned high-risk patients with severe AS who underwent either transcatheter aortic valve replacement with a balloon-expandable bovine pericardial valve or surgical replacement.^13^ The primary end-point was death from any cause at 1 year. The authors found that: 1) The rates of death from any cause were 3.4% in the transcatheter group and 6.5% in the surgical group at 30 days (*P* = 0.07) and 24.2% and 26.8%, respectively, at 1 year (*P* = 0.44), a reduction of 2.6 percentage points in the transcatheter group; 2) The rates of major stroke were 3.8% in the transcatheter group and 2.1% in the surgical group at 30 days (*P* = 0.20) and 5.1% and 2.4%, respectively, at 1 year (*P* = 0.07); 3) At 30 days, major vascular complications were significantly more frequent with transcatheter replacement (11.0% versus 3.2%, *P* < 0.001), and adverse events that were more frequent after surgical replacement included major bleeding (9.3% versus 19.5%, *P* < 0.001) and new-onset atrial fibrillation (8.6% versus 16.0%, *P* = 0.006); 4) More patients undergoing transcatheter replacement had an improvement in symptoms at 30 days, but by 1 year there was not a significant between-group difference. These key observations suggested that in high-risk patients with severe aortic stenosis, transcatheter and surgical procedures for aortic valve replacement were associated with similar rates of survival at 1 year, although there were important differences in periprocedural risks.

### The Implantation Techniques (Transfemoral, Transapical, Transaortic)

The first developed TAVI device is the Edwards SAPIEN valve (Edwards Lifesciences, Inc., Irvine, CA, USA). It consists of three bovine pericardial leaflets mounted within a balloon-expandable stainless-steel stent. Current prosthesis sizes include 23 and 26 mm. Current devices require either 22 F or 24 F (transfemoral) or 26 F (transapical) sheath for delivery. The Edwards SAPIEN valve was first implanted via the antegrade transseptal approach to the left atrium and passage through the mitral valve to reach the aortic valve (AV). With this approach there is a high risk of anterior mitral valve leaflet injury, causing severe mitral regurgitation. Transfemoral retrograde approach has been shown to be safer and is now preferred.^14^–^18^ Patients are usually placed under general anesthesia with endotracheal intubation, although sedation and analgesia may be sufficient. After crossing the AV, a balloon aortic valvuloplasty (BAV) is performed using standard techniques in order to pre-dilate the stenotic valve. Simultaneous rapid right ventricular pacing using a temporary pacemaker (usually 180 beats/min), decreasing cardiac output, is used to stabilize the balloon during the inflation.^19^ Because of the large profile of the device, many patients with small or diseased iliofemoral arteries are not eligible for the procedure or are at risk for major vascular complications. An alternative transapical antegrade approach has been proposed; through a left anterolateral minithoracotomy, with the patient under general anesthesia, the pericardium is opened over the apex. Temporary pacing wires are placed on the left ventricle (LV), the LV apex is punctured, and two pledgeted sutures are placed. A stiff wire is passed to the descending aorta, and BAV is performed. The percutaneous valve is then deployed.

As the number of patients screened for TAVI increases, many are found with absolutely no option for peripheral artery access. Therefore, Latsios et al. tested the safety and efficacy of the retrograde, minimally invasive, “transaortic” approach of transcatheter aortic valve implantation (TAVI) using the Medtronic CoreValve prosthesis (Medtronic, Minneapolis, MN, USA) as an alternative minimally invasive surgical access route.^20^ Two patients were carefully selected from a cohort of 580 patients: two women, aged 93 and 84 years, both with severe peripheral arterial occlusive disease. After a mini-sternotomy the ascending aorta was directly punctured. At the end, the access site was surgically sutured with the pre-positioned sutures. The patients were at all times off-pump and without intra-aortic balloon pump. The authors reported that TAVI was successful in both cases, leading to a fall in the transvalvular gradient with no cases of mortality, stroke, or myocardial infarction. The patients were extubated directly after the procedure, mobilized after 4 days, and were discharged home after 7 and 9 days thereafter. Hospitalization length was 34 days (patient #1) and 24 days (patient #2). These cases may support the notion that on rare occasions, where due to anatomical reasons transfemoral TAVI is not feasible, a minimally invasive “transaortic” approach, as described, provides an alternative option. This line of results follows the report by Bauernschmitt et al. who—based on a single case—also concluded that the “transaortic” approach might help to expand the implantation possibilities for those patients for whom the typical access sites are not available.^21^ However, the transapical TAVI is still the major alternative for the transfemoral approach due to pertinent potential advantages,^22^ including: 1) Lower rates of vascular complications, strokes, and use of contrast; 2) Larger sheath diameters which may lessen the need for crimping of the valves and thus improve longevity; and 3) Implementation of solutions for improving paravalvular leakage into clinical practice.

### TAVI in Octogenarians

In a recent study, Grimaldi et al. evaluated 145 octogenarians (aged 84.7 ± 3.4 years) who underwent TAVI for AS (97.2%) or isolated aortic regurgitation (2.8%).^23^ New York Heart Association (NYHA) class was 2.8 ± 0.6; Logistic EuroSCORE: 26.1 ± 16.7; Society of Thoracic Surgeons score: 9.2 ± 7.7. Echocardiographic assessments included aortic valve area (0.77 ± 0.21 cm^2^), mean/peak gradients (54.5 ± 12.2/88 ± 19.5 mmHg), left ventricular ejection fraction (LVEF) (21% of patients had an EF of less than 40%), systolic pressure in pulmonary artery (sPAP) (43.1 ± 11.6 mmHg). The main outcome measures of rates of mortality at 30 days, 6 months, and 1 year were 2.8%, 11.2%, and 17.5%, respectively. At 16-month follow-up, 85.5% survived showing improved NYHA class (2.8 ± 0.6 versus 1.5 ± 0.7, *P* < 0.001), decreased sPAP (43.1 ± 11.6 mmHg versus 37.1 ± 7.7 mmHg, *P* < 0.001), and increased LVEF in those with EF ≤ 40% (34.9 ± 6% versus 43.5 ± 14.4%, *P* = 0.006). Concerning QOL: 49% walked unassisted, 79% (39.5% among patients ≥ 85 years) reported self-awareness improvement; QOL was reported as “good” in 58% (31.4% among patients ≥ 85 years), “acceptable according to age” in 34% (16% among patients ≥ 85 years), and “bad” in 8%. These findings suggest TAVI procedures improve clinical outcome and subjective health-related QOL in elderly patients with symptomatic AS.

## BRAIN PROTECTION DURING CARDIAC SURGERY

Neurological injury is a significant risk for patients undergoing cardiac surgery, and it is associated with increased mortality, morbidity, hospital costs, and impaired quality of life.^24^ Cardiac surgery involves a wide spectrum of neurological injuries including ischemic stroke, occurring in 1.5% to 5.2% of patients, encephalopathy, affecting 8.4% to 32%, and neurocognitive dysfunction, manifested in 20% to 30% at 1 month post-surgery.^1^,^25^ Embolism is considered the main mechanism of neurological injury. Thirty to fifty percent of perioperative strokes detected with brain imaging are due to cerebral macroembolisms likely arising from the ascending aorta. Encephalopathy and neurocognitive dysfunction are believed to result primarily from cerebral microembolisms, which are either gaseous or particulate in composition. Gaseous emboli can arise from an open left-sided cardiac chamber or from air entrained into the cardiopulmonary bypass (CPB) circuit. An increased rate of embolic material released into the cerebral circulation has been detected during various aortic manipulations performed during cardiac surgery.

Several types of device that are used post-cannulation minimize the quantity of emboli released during cardiac surgery. First, proximal anastomotic devices are used to avoid partial aortic cross-clamping and minimize aortic manipulation during the proximal anastomosis. This approach is costly, requires advanced skill, and is prone to adverse surgical outcomes.^26^,^27^ Second, an ultrasound-based device that is placed on the aorta during surgery is used to divert the released particles from the cerebral circulation towards the descending aorta.^28^ Third, a percutaneously placed device inserted into the right forelimb and deployed in the aorta is performing a similar action of diversion of particles from the cerebral circulation.^29^ Finally, an intra-aortic filtration device incorporates an aortic cannula with a filter that captures particulate emboli during CPB.^30^

Yet another approach for intraoperative cerebral protection, which consists of deflecting embolic debris downstream in the aortic circulation, may also be associated with increased ischemic events within the systemic circulation, including the kidneys, gastrointestinal system, and lower extremities. The first example of this approach is a device that diverts the released particles from the carotid arteries to the descending aorta, using ultrasound waves. Notably, this device reduces embolic material in the cerebral circulation by approximately 50% compared to the greater reduction of 77% observed using the research cannula. The ultrasound device also suffers from adverse heating, which is potentially hazardous to involved tissues, varying operative routines, and a non-continuous mode of action, which leads to a narrowed removal of the embolic material from the systemic circulation.^28^ The second option is a percutaneously placed device inserted into the right forelimb and deployed in the aorta.^29^ As this device requires catheterization, it may be associated with vascular complications, a time-consuming course of action, costly procedures, and dissimilar operative routines.

A newly developed intraoperative cerebral protection device is the CardioGard cannula (CardioGard, Or-Yehuda, Israel), which simultaneously produces forward flow and backward suction to extract solid and gaseous emboli from the distal aorta as they are released during cardiac surgery. A preliminary animal study demonstrated that: 1) The CardioGard cannula yielded an overall embolus retrieval rate of 77%, with an 88.45% rate during a low-flow regimen used clinically during aortic manipulation; 2) Gaseous and solid emboli were eliminated by suction, as demonstrated by epicarotid ultrasound; and 3) No significant changes were observed in hemodynamic and laboratory parameters.^31^ In comparison to the aforementioned existing devices for intraoperative embolus reduction, the embolus removal quality of the CardioGard cannula allows the surgeon to perform the surgical procedure within safer limits of aortic manipulation, including cross-clamp placement and removal, as well as proximal anastomosis. The use of the intra-aortic filter device is more invasive and thus may be associated with unfavorable vascular complications such as plaque disruption during deployment and possible aortic perforation.^30^ Furthermore, the particle filtering cut-off value of the intra-aortic filter device is 120 mm in diameter, while that of the CardioGard cannula is 72 mm, corresponding to the external filter diameter. This dissimilarity may afford the double lumen cannula better embolus evacuation capacity. A human, multicenter, randomized, controlled clinical trial is currently under way.

## Abbreviations

AS: aortic stenosis;
AV: aortic valve;
AVR: aortic valve replacement;
BAV: balloon aortic valvuloplasty;
CABG: coronary artery bypass grafting;
CI: confidence interval;
CPB: cardiopulmonary bypass;
EuroSCORE: European System for Cardiac Operative Risk Evaluation;
LV: left ventricle;
LVEF: left ventricular ejection fraction;
MVR: mitral valve replacement;
NYHA: New York Heart Association;
OPCAB: off-pump coronary artery bypass;
OR: odds ratio;
QOL: quality of life;
sPAP: systolic pressure in pulmonary artery;
STS: Society of Thoracic Surgeons;
TAVI: transcatheter aortic valve implantation.
